# Virological Efficacy in Cerebrospinal Fluid and Neurocognitive Status in Patients with Long-Term Monotherapy Based on Lopinavir/Ritonavir: An Exploratory Study

**DOI:** 10.1371/journal.pone.0070201

**Published:** 2013-07-26

**Authors:** José R. Santos, José A. Muñoz-Moreno, José Moltó, Anna Prats, Adrià Curran, Pere Domingo, Josep M. Llibre, Daniel R. McClernon, Isabel Bravo, Jaume Canet, Victoria Watson, David Back, Bonaventura Clotet

**Affiliations:** 1 Lluita contra la SIDA Foundation, Hospital Universitari Germans Trias i Pujol, Barcelona, Spain; 2 Universitat Autònoma de Barcelona, Barcelona, Spain; 3 Infectious Diseases Department, Hospital Vall d’Hebron, Barcelona, Spain; 4 Infectious Diseases Unit, Hospital de la Santa Creu i Sant Pau, Barcelona, Spain; 5 bioMONTR, Research Triangle Park, North Carolina, United States of America; 6 Department of Anesthesiology and Postoperative Care Unit, Hospital Universitari Germans Trias i Pujol, Barcelona, Spain; 7 Department of Molecular and Clinical Pharmacology, University of Liverpool, Liverpool, United Kingdom; 8 Bioanalytical Facility, Royal Liverpool Hospital Trust, Liverpool, United Kingdom; 9 IrsiCaixa Foundation, Barcelona, Spain; Temple University School of Medicine, United States of America

## Abstract

**Background:**

Data on suppression of HIV replication in the CNS and on the subsequent risk of neurocognitive impairment using monotherapy with boosted protease inhibitors are limited.

**Methods:**

Ours was an exploratory cross-sectional study in patients on lopinavir/ritonavir-based monotherapy (LPV/r-MT) or standard triple therapy (LPV/r-ART) for at least 96 weeks who maintained a plasma viral load <50 copies/mL. HIV-1 RNA in CSF was determined by HIV-1 SuperLow assay (lower limit of detection, 1 copy/mL). Neurocognitive functioning was assessed using a recommended battery of neuropsychological tests covering 7 areas. Neurocognitive impairment (NCI) was determined and also a global deficit score (GDS) for study comparisons.

**Results:**

Seventeen patients on LPV/r-MT and 17 on LPV/r-ART were included. Fourteen (82.4%) patients on LPV/r-MT and 16 (94.1%) on LPV/r-ART had HIV-1 RNA <1 copy/mL in CSF (p = 0.601). NCI was observed in 7 patients on LPV/r-MT and in 10 on LPV/r-ART (41% vs 59%; p = 0.494). Mean (SD) GDS was 0.22 (0.20) in patients on LPV/r-MT and 0.47 (0.34) in those on LPV/r-ART (p = 0.012).

**Conclusions:**

Suppression of HIV in CSF is similar in individuals with durable plasma HIV-1 RNA suppression who are receiving LPV/r-MT or LPV/r-ART for at least 96 weeks. Findings for HIV-1 replication in CSF and neurocognitive status indicate that this strategy seems to be safe for CNS functioning.

## Introduction

Standard-of-care antiretroviral therapy (ART) is based on a combination of at least 3 antiretroviral drugs including 2 nucleos(t)ide reverse transcriptase inhibitors (NRTIs) plus 1 non-nucleoside reverse transcriptase inhibitor (NNRTI), 1 boosted-protease inhibitor (PI), or 1 integrase inhibitor [1]–[3]. However, NRTIs can cause kidney and bone toxicity and mitochondrial dysfunction, which may in turn be associated with side effects [4]–[6]. It is thus advisable to investigate therapeutic strategies that avoid prolonged exposure to NRTIs and their side effects.

Monotherapy with boosted PIs is particularly attractive as an NRTI-sparing strategy. Guidelines consider this approach in cases of NRTI-related toxicity or intolerance [1], [2], [7]. In addition, PI monotherapy could improve adherence, decrease costs, and preserve future treatment options [8]–[10]. According to data obtained from clinical trials and routine clinical practice, simplification to lopinavir/ritonavir (LPV/r) monotherapy in well-suppressed HIV-1-infected patients seems to be as effective as triple ART for maintaining virological suppression when resuppression of HIV-1 RNA by reintroducing NRTIs is not considered failure [11]–[13].

Nonetheless, consequences of PI monotherapy on the HIV-1 compartments have not been well studied. One of the concerns with monotherapy is whether it can maintain control of viral replication in different compartments, particularly in cerebrospinal fluid (CSF), where it has been associated with a higher risk of neurocognitive impairment by some authors [14]. Although LPV/r has relatively high CSF penetration compared with other antiretroviral drugs [15], [16], its Central Nervous System Penetration-Effectiveness (CPE) score remains lower than that of standard triple ART [15]. Moreover, additional data suggest that LPV/r monotherapy in CSF may be inadequate to maintain suppression of viral replication in that compartment [17]. Consequently, the virological efficacy of LPV/r monotherapy in CSF and its possible consequences have been questioned.

The objective of this study was to explore the long-term virological efficacy of LPV/r monotherapy in CSF and neurocognitive function in patients with HIV-1 infection and sustained suppression of plasma viral load.

## Methods

### Study Design and Patients

We performed an exploratory, comparative, cross-sectional study of patients with HIV-1 infection and stable antiretroviral treatment with either LPV/r in monotherapy (LPV/r-MT) or LPV/r plus 2 NRTIs (LPV/r-ART) for at least 96 weeks, while maintaining a plasma viral load <50 copies/mL to assess a complete virological suppression in CSF (CSF viral load <1 copy/mL).

This study was conducted by the Lluita contra la Sida Foundation, Barcelona, Spain between August 2010 and June 2011. From a prospectively compiled database (electronic medical files), we identified all consecutive patients who were receiving stable treatment with LPV/r at doses of 400/100 mg twice daily (Kaletra®, Abbott Laboratories, Abbott Park, Illinois, USA) in monotherapy or in combination with two NRTIs. The exclusion criteria were adherence <90%, temporary discontinuation of LPV/r for any reason, or medical contraindications for lumbar puncture during the current regimen. Patients with potential confounding comorbidities for the existence of neurocognitive impairment were included, not only to obtain a representative clinical sample of patients with HIV, but also to consider this variable in data comparisons. Confounding factors included self-reported past or current disease involving the CNS, diagnosis of a psychiatric disorder, psychopharmacologic treatment, or active/past drug use.

The primary endpoint was the percentage of patients with complete virological suppression in CSF after 96 weeks on LPV/r-MT. Secondary endpoints were the percentage of patients with neurocognitive impairment, differences in neurocognitive status in terms of the global deficit score (GDS), and the assessment of LPV trough concentrations in CSF and plasma.

The study was approved by the ethics committee of Hospital Germans Trias i Pujol (NCT 01116817), Badalona, Spain and performed according to the stipulations of the Declaration of Helsinki (Seoul, 2008). All patients gave their written informed consent before enrollment.

### Study Procedures

We recorded demographic and clinical variables including age, gender, ethnicity, nadir CD4+ T-cell count, current CD4+ T-cell count, CDC stage, hepatitis C virus co-infection, zenith of HIV-1 viral load, time since diagnosis of HIV infection, time with virological suppression (HIV-1 RNA <50 copies/mL), time on antiretroviral treatment, time on LPV/r-MT or LPV/r-ART, history of opportunistic infections and other comorbidities, and concomitant medication. Patients recorded the time they had taken the last LPV/r dose on the day before the visit.

Paired CSF-plasma samples were obtained from each participant on the day of the study visit, and time of sampling was recorded. Plasma HIV-1 RNA, CD4+ T-cell count, and routine hematology and biochemistry tests were performed at the local laboratory.

The method used for ultrasensitive measurement of HIV-1 RNA in CSF was the HIV-1 SuperLow assay. This method is based on a proprietary protocol and algorithm (bioMONTR®) [18], which is used in conjunction with a CE-marked commercial HIV-1 RNA EasyQ reagent kit (bioMérieux, Inc, Lyon, France). A specimen of 1–2 mL of human CSF was added to lysis buffer containing guanidine thiocyanate. HIV-1 RNA was extracted using a propriety protocol (bioMONTR®) [18] in combination with the EasyMAG platform (bioMérieux, Inc). Eluates containing HIV-1 RNA was aliquoted into 0.5-mL reaction tubes and amplified using 3 enzymes: T7 RNA polymerase, avian myeloblastosis virus reverse transcriptase, and RNase H. Primer. Molecular beacons targeting the pol/gag region of HIV-1 RNA were utilized for amplification and detection by isothermal reactions at 41°C. HIV-1 viral load was quantified using a proprietary reduction algorithm (bioMONTR®) [18] in conjunction with the NucliSENS EasyQ® HIV-1 v2.0 Director software. The dynamic range of the assay was 1–5,000,000 copies/mL. Assay sensitivity was adjusted for the volume of CSF available from each patient.

LPV concentrations in CSF were quantified using a validated protein precipitation method coupled with reverse-phase liquid chromatography-tandem mass spectrometry. CSF samples (100 µL) were extracted via protein precipitation (acetonitrile: 0.1% formic acid [50∶50] 200 µL) with the addition of an internal standard (Quinoxaline [QX] 1 µg/mL; 20 µL). LPV and QX were resolved on a reverse-phase Ascentis C_18_ column (3 µm: 100 mm×2.1 mm) using a step-wise gradient mobile phase. LPV was quantified using a triple-quadrupole mass spectrometer (TSQ Access Max, Thermo Scientific, UK). A 9-point linear CSF calibration curve (range, 1.02–78.38 ng/mL) was validated using artificial CSF (Harvard Apparatus, UK) with the addition of human serum albumin (0.2 g/L) (Sigma, UK). Accuracy ranged from 101.2–104% and imprecision was <8%. The recovery rate was high and reproducible.

Blood samples for determination of LPV concentrations in plasma were collected in potassium EDTA tubes. Plasma was isolated by centrifugation (1500×*g*, 15 minutes) and stored at –20°C until analysis. LPV concentrations were determined using high-performance liquid chromatography with a photo diode array detector (HPLC-PDA 2996; Waters Corporation, Barcelona, Spain) according to a validated method, which involved liquid-liquid extraction with tert-butyl methyl ether. The mobile phase consisted of gradient elution with phosphate buffer-acetonitrile. The method was linear over the range of 0.05–20 mg/L. Intra- and interday coefficients of variation were less than 10%. The assay was externally validated using the International Interlaboratory Quality Control Program for Therapeutic Drug Monitoring in HIV Infection (KKGT, Nijmegen, the Netherlands) [19].

### Neurocognitive Assessment

Neurocognitive status was assessed using a comprehensive neuropsychological battery of 15 tests that covered 7 areas recommended for evaluation in HIV infection [20], [21]. The tests included and the areas assessed were as follows: the Letter-Number Sequencing and Digit Span Tests of the Wechsler Adult Intelligence Scale-III (WAIS-III) [22] for attention/working memory; Part A of the Trail Making Test (TMT) [23] and the Symbol Digit Modalities Test (SDMT) [24] for information processing speed; the California Verbal Learning Test (CVLT) [25] for verbal memory and learning; Part B of the TMT [23], the Stroop Test [26], the Wisconsin Card Sorting Test (WCST) [27], and the Tower of London (TOL) test [28] for executive function; the Controlled Oral Word Association Test (COWAT) [29] and the Animals Test [30] for verbal fluency; and the Grooved Pegboard Test [31] for motor function. Premorbid intelligence was also assessed using the Vocabulary Test of the WAIS-III [22]. The Frascati criteria proposed by Antinori et al [20]. were used to classify subjects in both groups according to the presence of HIV-associated neurocognitive disorders (HAND). This classification took into account the presence of asymptomatic neurocognitive impairment (ANI), mild neurocognitive disorder (MND), and HIV-associated dementia (HAD) and was applied to describe the characteristics of the sample in terms of the specific HAND presented. Standardized T scores were used for comparison of neurocognitive outcomes and calculated by a converting process based on adjusting the raw scores according to normative data. This adjustment covered principally age, gender, and education level, according to available published data [25], [28]–[30], [32]–[36]. Categorically, neurocognitive impairment was defined as performing at least 1 standard deviation below the normative mean on at least 2 areas according to the T scores. Quantitative comparisons between groups were made using the GDS, which is a validated sensitive method to study differences in neurocognitive status [37]. Emotional status was assessed based on symptoms of depression and anxiety. The Beck Depression Inventory (BDI) was used to evaluate depressive symptoms, and the State-Trait Anxiety Inventory was used to evaluate anxiety symptoms [38], [39].

### Statistical Analysis

Patients receiving either LPV/r-MT or LPV/r-ART were matched according to the following characteristics: age, gender, CD4+ T-cell count at enrollment, nadir CD4+ T-cell count, and time on LPV/r-based treatment. Variables with a normal distribution were described as mean (standard deviation [SD]) and compared using the *t* test. Median (interquartile range [IQR]) was used to describe non-normally distributed variables, which were compared using the Mann–Whitney test. Percentages were compared using the χ*^2^* square test. Because this was an observational and cross-sectional investigation, the possibility of a lack of equivalence between groups was considered, and neuropsychological measures were adjusted using linear and logistic regressions when demographic or clinical variables were not balanced. All comparisons and statistical analyses were performed using SPSS version 15.0 (Chicago, Illinois, USA). Differences were considered statistically significant at *p*<0.05.

## Results

### Study Sample Characteristics

Thirty-seven patients agreed to participate in the study. Of these, 3 were excluded because they did not fulfill the inclusion criteria. Therefore, the final sample comprised 34 patients (17 on LPV/r-MT and 17 on LPV/r-ART): 29 (85.3%) were male, and the median (IQR) age was 47.3 (41.4–50.0) years. Overall, the median time on antiretroviral treatment was 8.1 (5.5–17.2) years and the median time on LVP/r-based treatment and with HIV-1 RNA <50 copies/mL was 3.8 (2.5–4.7) and 5.3 (3.5–7.4) years, respectively. The median number of previous antiretroviral regimens for patients receiving LPV/r-MT and patients receiving LPV/r-ART was 6 (2–10) and 2 (1–4), respectively (p = 0.018). Median time with virological suppression was 6.9 (5.5–8.9) years and 3.4 (2.3–5.1) years (p<0.001). The remaining demographic and clinical variables were similar for both groups (Table 1).

**Table 1:** Demographic and clinical characteristics^a^.

	LPV/r-MT	LPV/r-ART	P value
Age (years)	45.2 (38.9–48.7)	47.3 (42.9–50.1)	0.547
Male	15 (88.2)	14 (82.4)	0.628
MSM	10 (58.8)	7 (41.2)	0.294
Years of education	12 (9–17)	9 (8–12)	0.060
Employed	13 (76)	13 (76)	1.000
CDC stage C	2 (11.8)	3 (17.6)	0.064
HCV co-infection	9 (52.9)	3 (17.6)	0.071
CD4+ T-cell nadir (cells/mm^3^)	186 (118–294)	169 (61–293)	0.744
Prior ARV regimens	6 (2–10)	2 (1–4)	0.018
Prior NNRTIs	1 (0–2)	0 (0–1)	0.085
Prior PIs	2 (1–3)	1 (1–2.5)	0.401
Prior NRTIs	5 (3–6)	3 (2–5)	0.164
Time since diagnosis of HIV (years)	17.1 (8.3–20.4)	8.7 (5.0–18.1)	0.076
Time on treatment (years)	10.6 (6.1–17.9)	7.3 (3.2–14.8)	0.088
Time with virological suppression (years)	6.9 (5.5–8.9)	3.4 (2.3–5.1)	<0.001
Time on LPV/r-based treatment (years)	3.8 (2.7–4.8)	3.7 (2.4–4.7)	0.524
Current NRTI backbone			
TDF+FTC	–	14 (82)	–
ABC +3TC	–	2 (12)	–
AZT+ddI	–	1 (6)	–
Zenith VL (log)	4.8 (3.8–5.5)	4.9 (4.5–5.4)	0.564
CD4+ T-cell count (cells/mm^3^)	736 (579–856)	570 (419–818)	0.085
Premorbid intelligence (WAIS-III Vocabulary Test)^b^	56 (50–62)	51 (44–56)	0.192
Depression (BDI)^b^	55 (45–61)	57 (51–60)	0.572
Anxiety (STAI)^b^	56 (45–62)	55 (49–61)	0.802
Patients with confounding comorbidities^c^	4 (23)	4 (23)	1.000

a Values are expressed as No. (%) or median (interquartile range).

b Standardized T scores based on normative data.

c Previous or current disease involving the CNS, psychiatric disorder, psychopharmacologic treatment, and drug use.

Abbreviations: MSM, men who have sex with men; CDC, Centers for Disease Control and Prevention; ARV, antiretroviral; PIs, protease inhibitors; NRTIs, nucleoside reverse transcriptase inhibitors; NNRTIs, nonnucleoside reverse transcriptase inhibitors; LPV/r, lopinavir/ritonavir; LPV/r-MT, lopinavir/ritonavir monotherapy; LPV/r-ART, lopinavir/ritonavir triple-therapy; TDF, tenofovir; FTC, emtricitabine; ABC, abacavir, 3TC, lamivudine; AZT, zidovudine; ddI, didanosine; VL, viral load; WAIS-III, Wechsler Adult Intelligence Scale-III; Beck Depression Inventory; STAI, State-Trait Anxiety Inventory.

Three patients presented adverse events related to study procedures. Two patients experienced mild headache after lumbar puncture, and 1 patient had pain at the lumbar site during the 7 days after lumbar puncture.

### Virological Outcomes

Fourteen (82.4%) patients on LPV/r-MT and 16 (94.1%) on LPV/r-ART had HIV-1 RNA <1 copy/mL in CSF (p = 0.601). Among patients who had a CSF HIV-1 RNA ≥1 copy/mL, 3 patients on LPV/r-MT had determinations of 1, 75, and 120 copies/mL, respectively, and the patient on LPV/r-ART (LPV/r plus abacavir/lamivudine co-formulation) had an HIV-1 RNA of 2 copies/mL.

### Neurocognitive Outcomes

**Figure 1:**
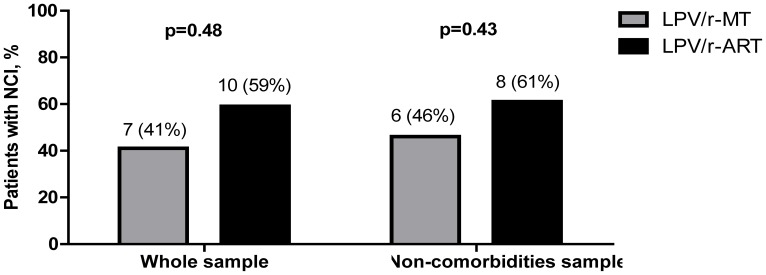
Percentages of patients with neurocognitive impairment. Abbreviations: LPV/r-MT, lopinavir/ritonavir monotherapy; LPV/r-ART, LPV/r triple-therapy; NCI, neurocognitive impairment. Comorbidities: depression or anxiety, drug use, psychiatric diagnosis, psychopharmacologic treatment.

Neurocognitive impairment was observed in 7 patients on LPV/r-MT and 10 patients on LPV/r-ART (41% vs 59%; p = 0.494). When patients with potential confounding comorbidities for neurocognitive impairment were excluded, the results were similar: 6/13 (46%) patients receiving LPV/r-MT and 8/13 (61%) patients receiving LPV/r-ART had neurocognitive impairment (p = 0.43) (see Figure 1). According to the HAND classification [20], all patients with neurocognitive impairment in the LPV/r-MT group had an ANI; in the LPV/r-ART group, 4 (50%) individuals had an ANI and 4 (50%) an MND.

Significant differences were observed between groups in neurocognitive functioning. The median (IQR) GDS was 0.2 (0.07–0.36) in the LPV/r-MT group and 0.47 (0.27–0.67) in the LPV/r-ART group (p = 0.012). When patients with potential comorbidities were not included in the analysis, differences were mostly similar: median (IQR) GDS was 0.27 (0.03–0.43) in the LPV/r-MT group and 0.47 (0.27–0.73) in the LPV/r-ART group (p = 0.022). Adjusted analyses with linear and logistic regressions confirmed that years of education, CDC stage, HCV co-infection, time since diagnosis of HIV, prior antiretroviral regimens and time with virological suppression were not associated with changes in neurocognitive impairment and GDS.

### Lopinavir Concentrations in CSF and Plasma

Median (IQR) time since the last dose was 12.8 (9.8–14.7) hours. Median plasma and CSF LPV concentrations were 5083 (4100–8562) ng/mL and 22.3 (10.7–31.4) ng/mL, respectively. Two plasma LPV samples and one CSF sample were below the lower limit of quantification (<0.05 mg/L and <1.02 ng/mL, respectively), suggesting poor recent adherence. Overall, LPV concentrations in plasma and CSF were well correlated (rho = 0.723, p<0.001). The median (IQR) ratio of CSF to plasma concentration was 0.46% (0.34%–0.75%). LPV concentrations in CSF exceeded the 50% inhibitory concentration (IC_50_) for wild-type HIV-1 (0.69 ng/mL; no adjustment for protein binding) [40] by a median (IQR) of 32.2–fold (15.5–45.6). A LPV concentration in CSF below 0.69 ng/mL was recorded in only 1 patient who had detectable HIV-1 RNA in CSF.

## Discussion

In patients taking stable antiretroviral treatment with LPV/r-ART or LPV/r-MT and with maintained plasma HIV-RNA <50 copies/mL for at least 96 weeks, the percentage of patients with complete virological suppression in CSF was similar in both groups. In addition, the proportion of patients with neurocognitive impairment was also similar between the groups. Therefore, our data suggest that HIV replication in CSF is suppressed with similar efficacy with LPV/r MT and LPV/r ART in individuals with long-term suppression (96 weeks) of plasma HIV-1 RNA.

Although up to 99% of LPV binds to proteins in plasma, LPV concentrations in CSF exceed the median inhibitory concentration for wild-type HIV-1 after boosting with ritonavir at standard doses [41], [42]. This explains the high CPE rank assigned to LPV/r in comparison with other antiretroviral drugs and the capacity of this combination to suppress viral replication in CSF [16], [42], [43], despite its CPE score remains lower than that of standard triple ART [15]. All but 1 patient in our study had LPV concentrations in CSF above the IC_50_ for wild-type strains of HIV. This finding, together with the similar rates of complete CSF-virological suppression in patients receiving long-term LPV/r-MT and LPV/r-ART, support monotherapy LPV being able to maintain virological suppression in CSF, even taking into account that most of patients included in the group of LPV/r ART had TDF/FTC as backbone.

CPE has been proposed as a clinical tool to evaluate the penetration of antiretroviral drug combinations in the CSF of patients with standard triple ART [43]. The CPE rank of LPV/r monotherapy is lower than that of other triple therapy combination based on LPV/r, although it has been designed to compare similar triple ART-based regimens and not validated with PI monotherapy. Therefore, reasonable doubts have arisen about the ability of LPV/r monotherapy to maintain virological suppression in CSF. In addition, the results of the MOST study stated that monotherapy with LPV/r might be insufficient to control virological replication in CSF [17]. The conclusions, however, were based on patients who had experienced virological failure in plasma, with subsequent elevation of HIV-1 RNA in CSF and development of neurological symptoms [44]. In our series, the percentage of patients with complete virological suppression in CSF and neurocognitive impairment was similar between groups. Neurocognitive functioning in terms of GDS was even slightly better in patients on LPV/r-MT than in patients on triple LPV/r-ART. The better GDS results in patients on LPV/r-MT may be in part explained by the limited sample size and the fact that only patients virologically suppressed for at least 96 weeks were selected. These findings, however, are consistent with those of Marra et al. [45], who observed poorer neurocognitive performance in patients with higher CPE scores than those receiving regimens with lower CPE scores, thus raising the issue of potential neurologic toxicity of antiretroviral therapy. In addition, Bunupuradah et al [46]. recently reported similar findings for patients who started LPV/r monotherapy after non–NRTI-based ART had failed. Finally, our results are also concordant with long-term follow-up data from a clinical trial that showed the absence of neurological adverse events [47], thus suggesting that monotherapy with a boosted PI alone is not a determining factor for the development of neurocognitive impairment.

Our study is subject to a series of limitations. First, the sample was small, and the exploratory cross-sectional design could lead to bias or unmeasured confounding factors. Second, selection bias is implicit with the inclusion of only those patients who had treatment for at least 96 weeks with maintained HIV virological suppression. Therefore, we were not able to evaluate those patients who could have developed neurocognitive impairment before the study was performed. In addition, as mentioned above, selection bias could also have affected the quantitative difference observed in neurocognitive functioning between groups. Third, despite the fact that patients were matched for the main demographic and immunological characteristics, differences were observed. Patients on LPV/r monotherapy had previously used significantly more antiretroviral regimens and had more time with virological suppression, although both groups had similar time on antiretroviral treatments, partly because patients on LPV/r-MT had initiated monotherapy due to poor tolerance, toxicity, and simplification. Adjusted analyses, however, confirmed that there were no association between these variables and neurocognitive performance. In addition, patients on LPV/r-MT showed a non-significant trend to higher CD4+ T-cell count and more years of education at inclusion, which also suggests that monotherapy with LPV/r was used in more selected patients. Despite these limitations, ours is the first study to explore long-term virological efficacy in CSF and neurocognitive safety of LPV/r-MT as an NRTI-sparing strategy in patients with HIV-1 infection and sustained plasma virological suppression. Cohort and clinical trials with specific neurocognitive endpoints are ongoing, although results are not anticipated in the short or medium term [48], [49]. Results of these studies should be important to confirm our results and to evaluate prospectively the possible consequences of this strategy on the neurocognitive performance.

In conclusion, the results of sensitive CSF examination and neurocognitive performance suggest that suppression of HIV-1 RNA is similar in patients with HIV-1 RNA suppression receiving monotherapy with LPV/r and patients receiving LPV/r-based ART for at least 96 weeks. However, our findings should be confirmed in prospective randomized trials.
